# Plasma Lipid Profiling of Patients with Chronic Ocular Complications Caused by Stevens-Johnson Syndrome/Toxic Epidermal Necrolysis

**DOI:** 10.1371/journal.pone.0167402

**Published:** 2016-11-29

**Authors:** Kosuke Saito, Mayumi Ueta, Keiko Maekawa, Chie Sotozono, Shigeru Kinoshita, Yoshiro Saito

**Affiliations:** 1 Division of Medical Safety Science, National Institute of Health Sciences, Setagaya, Tokyo, Japan; 2 Department of Frontier Medical Science and Technology for Ophthalmology, Kyoto Prefectural University of Medicine, Kamigyo-ku, Kyoto, Japan; 3 Department of Ophthalmology, Kyoto Prefectural University of Medicine, Kamigyo-ku, Kyoto, Japan; Pacific Northwest National Laboratory, UNITED STATES

## Abstract

Stevens-Johnson syndrome (SJS) and its severe variant, toxic epidermal necrolysis (TEN), are drug-induced acute inflammatory vesiculobullous reactions of the skin and mucous membranes, including the ocular surface. Even after recovery from skin symptoms, some SJS/TEN patients continue to suffer with severe ocular complications (SOCs). Therefore, this study aims to understand the pathophysiology of chronic SOCs. Because plasma lipid profiling has emerged as a useful tool to understand pathophysiological alterations in the body, we performed plasma lipid profiling of 17 patients who suffered from SJS/TEN-associated chronic SOCs. A lipidomics approach yielded 386 lipid molecules and demonstrated that plasma levels of inflammatory oxylipins increased in patients with SJS/TEN-associated chronic SOCs. In addition, oxidized phosphatidylcholines and ether-type diacylglycerols increased in the patients with chronic SOCs, while phosphoglycerolipids decreased. When we compared these lipidomic profiles with those of patients with atopic dermatitis, we found that patients with chronic SOCs, specifically, had decreased levels of ether-type phosphatidylcholines (ePCs) containing arachidonic acid (AA), such as PC(18:0e/20:4) and PC(20:0e/20:4). To confirm our finding, we recruited additional patients, who suffered from SOC associated with SJS/TEN (up to 51 patients), and validated the decreased plasma levels of AA-containing ePCs. Our study provides insight into the alterations of plasma lipidomic profiles in chronic SOCs and into the pathophysiology of SJS/TEN-associated chronic SOCs.

## Introduction

Stevens-Johnson syndrome (SJS) and its severe variant, toxic epidermal necrolysis (TEN), are drug-induced acute inflammatory vesiculobullous reactions of the skin and mucous membranes, including the ocular surface [1,2]. Although the annual incidence rates of SJS/TEN are low (estimated as 1–6/0.4–1 cases per million persons, respectively) [3], their mortality rates were 3% and 27% of patients, respectively [4]. Approximately 40% of SJS/TEN patients develop severe ocular complications (SOCs), severe conjunctivitis with ocular surface epithelial defects and pseudomembrane, in the acute stage [5,6]. Cold medicines (CMs), such as acetaminophen, are drugs commonly suspected of causing SOCs associated with SJS/TEN, and genetic polymorphisms play some role [7–9]. For example, our recent work has demonstrated that HLA-A*02:*06* and -B*44:*03* were associated with the incidence of SOCs in CM-related SJS/TEN patients [10]. On the other hand, even after recovery from skin symptoms, some SJS/TEN patients continue to suffer with SOCs [11], and sometimes the ocular condition worsens due to inflammation. Therefore, chronic SOCs is one of the major conditions observed in SJS/TEN patients.

Lipids, such as oxylipins, phosphoglycerolipids, sphingolipids, and neutral lipids, play important roles in multiple biological processes, including apoptosis, proliferation and differentiation, as well as the inflammatory response. For example, oxylipins, such as prostaglandins, including prostaglandin E_2_ (PGE_2_), and leukotrienes play a pivotal role in the inflammatory response [12,13]. In addition, ceramides (Cer), a class of sphingolipids, can accumulate and mediate inflammation [14]. Moreover, glycosylceramide (GCer) and ether-type lysophosphatidylethanolamine (eLPE) have been reported to act as antigens presented by CD1d against invariant natural killer T (iNKT) cells [15,16]. Thus, it is possible that alterations in lipid profiles could be associated with pathophysiological changes in SJS/TEN patients with chronic SOCs. Our observation of down-regulation of PGE_2_ receptor 3 (EP3) in the conjunctival epithelium of SJS/TEN patients would also support this possibility [7].

Recently, lipidomics, an approach to obtaining an overview of the lipid profile, has been established using a platform combined with liquid chromatography and mass spectrometry [17,18]. Using this approach, plasma lipid profiling has emerged as a useful tool to understand the pathophysiological alterations in lipids in specific organs. For example, plasma lipid profiling revealed that lysophosphatidylcholines (LPCs), such as LPC(14:0), LPC(16:0), and LPC(18:0), are down-regulated in patients with hepatocellular carcinoma [19]. In addition, aberrant plasma lipid profiles were found in prediabetes patients as well as in patients with type 2 diabetes [20]. Moreover, lipid profiling revealed that lipoxygenase (LOX) metabolites are involved in both inflammation and resolution of inflammation induced by influenza infection [21]. Although lipidomics approach has not yet been applied for plasma lipid profiling of patients with SJS/TEN, it is known that the skin lipid profile is altered in patients who suffered from atopic dermatitis [22]. Therefore, we hypothesized that plasma lipid profiling using lipidomics would help illuminate the pathophysiological mechanisms of SJS/TEN-associated chronic SOCs.

In the present study, we first collected plasma samples from SJS/TEN patients suffering from chronic SOCs. For the screening phase, we performed lipid profiling using a lipidomics approach and compared the profiles of healthy volunteers with those of SJS/TEN-associated chronic SOC patients or atopic dermatitis patients, another inflammatory disease. The lipidomics approach provided an overview of 386 lipids, including 114 phosphoglycerolipids, 47 sphingolipids, 182 neutral lipids, and 43 oxylipins. Once we identified the specific lipids altered in chronic SOCs, we calculated the optimal sample size for reliable statistical power based on effect size, and collected samples from additional subjects to validate our findings. Here, we present novel alterations in lipid profiles in patients with chronic SOCs compared to lipid profiles observed in healthy volunteers. Our study provides useful information addressing pathophysiological mechanisms that are potential therapy targets for SJS/TEN-associated chronic SOCs.

## Methods

### Patients and plasma samples

This study was approved by the institutional review boards of Kyoto Prefectural University of Medicine (KPUM) and the National Institute of Health Sciences. All experimental procedures were conducted in accordance with the principles of the Helsinki Declaration and all patients included in this study provided written informed consent.

Because ophthalmologists usually encounter SJS/TEN patients in the chronic rather than the acute stage, many of our patients had developed SJS/TEN many years before their recruitment for this study. The diagnosis of SJS/TEN was based on a confirmed history of acute-onset high fever, serious mucocutaneous illness with skin eruptions, and involvement of at least two mucosal sites, including the ocular surface.

We defined patients with SOCs as patients with ocular sequelae, such as severe dry eye, symblepharon, trichiasis, and conjunctival invasion into the cornea in the chronic stage. We recruited 17 patients with CM-related and SJS/TEN-associated chronic SOCs, in addition to 32 healthy volunteers and 24 atopic dermatitis patients at KPUM, who acted as controls for the screening study. Of the 17 patients with CM-SJS/TEN with SOCs, 5 were males and 12 were females; their ages ranged from 17 to 92 years (average 49.7 ± 19.0 (SD) years). The normal controls (healthy volunteers, 10 males and 22 females) had a median age of 40.3 ± 10.6 years. The 24 atopic dermatitis patients (18 males and 6 females), who represented inflammatory disease controls, had a median age of 23.9 ± 15.3 years. Once specific lipids were identified, we further recruited up to 55 patients with CM-related and SJS/TEN-associated chronic SOCs and 51 healthy volunteers for the validation study. The characteristics of the subjects recruited in the validation study are summarized in Table 1. Blood samples were collected using tubes with EDTA-2Na as a coagulant; the tubes were centrifuged, and then plasma was isolated. Because all subjects were recruited in the department of ophthalmology, the only information available was the age and sex of subjects.

**Table 1:** Information of subjects recruited in the validation study.

	Healthy	SOCs
Number of subjects	55	51
Age range	21–63	7–92
Age average ± SD	40.5 ± 10.7	49.1 ± 18.1**
Male	20	18
Female	35	33
Age (20 to 55)	47	29
Age range (20 to 55)	21–54	23–55
Age average ± SD (20 to 55)	37.4 ± 8.3	41.6 ± 10.8^ns^

**; p < 0.01 between healthy volunteers and patients with chronic SOCs.

ns; not statistically significant

### Lipid extraction

For the screening study, lipid extraction from plasma was performed as described previously [23]. For the validation study, lipids were extracted from 40 μL of plasma. Four times the volume of methanol containing an internal standard (diether-PC[12:0/12:0]) was added, vortexed for 1 min, centrifuged 12000 *g* for 4 min, and filtered. The resulting solution (corresponding to 1 μL of plasma) was used for the quantification of AA-containing PCs altered in patients with SJS/TEN-associated chronic SOCs.

### Measurement of phosphoglycerolipids, sphingolipids, and neutral lipids in the screening study

Phosphoglycerolipids, sphingolipids, and neutral lipids were measured using chromatography-time-of-flight mass spectrometry (LC/TOFMS; ACQUITY UPLC System [Waters, Milford, MA]/LCT Premier XE [Waters]), as described previously [23]. The plasma samples of patients with SJS/TEN-associated chronic SOCs, patients with atopic dermatitis, and healthy volunteers were randomized across the run. In addition, extracted ion peaks were further analyzed to identify lipid molecules as described previously [23]. Processing of extracted ion peaks yielded 343 lipid molecules (161 and 182 lipid molecules from negative and positive ion mode, respectively) (S1 Table). We put alphabet, “a”, “b” or “c”, at the end of lipid molecules to distinguish lipid molecules with the same formula discriminated by retention time.

### Measurement of oxylipins in the screening study

Oxylipins were measured by a targeted approach using LC/MS/MS (ACQUITY UPLC System/5500QTRAP quadrupole-linear ion trap hybrid mass spectrometer [AB Sciex, Framingham, MA]), as described previously [23]. Because several oxylipins, such as leukotriene C and 15-hydroperoxy eicosatetraenoic acid, are unstable and break down during blood circulation and sample preparation, they were not targeted in the present study. The plasma samples of SJS/TEN patients, atopic dermatitis patients and healthy volunteers were randomized across the run. The processing of targeted lipid molecules yielded 43 lipid molecules (S1 Table).

### Lipidomics data processing of the screening study

The data cut off point of measurement of phosphoglycerolipids, sphingolipids, and neutral lipids was set at a height of 50 for negative ion mode and 100 for positive ion mode, whereas the measurement of oxylipins used height and SN ratios that were set at 1000 and 10, respectively. For samples with missing values for a metabolite, the cut off value was applied as the values for measurement of phosphoglycerolipids, sphingolipids, and neutral lipids, and the minimum observed value of the metabolites among all samples was applied as the missing values for the measurement of oxylipins. To correct variations in each sample run, the intensities of each extracted ion peak were normalized to those of the internal standard (PC[16:0/16:0-d6]) in the measurement of phosphoglycerolipids, sphingolipids, and neutral lipids. For the measurement of oxylipins, the areas of each ion peak from lipid molecules were normalized to those of the internal standard (LTB_4_-d4). The RSD of the internal standard (PC[16:0/16:0-d6] in negative and positive ion mode and LTB_4_-d4), which monitors experimental quality throughout the extraction, measurement, and data processing were 10.0%, 9.3%, and 13.6%, respectively. All data were presented as relative levels to the median values of all measured samples. Significant differences in the metabolite levels were assessed by Welch’s *t*-test. In this study, p < 0.05 represents statistical significance for all measurements. The processed data, average values, and standard deviations of the levels of lipid molecules as well as statistical values (p value) and false discovery rate (FDR) are presented in S2–S4 Tables. The effect sizes and the required total sample size for α = 0.05 and β = 0.1 were calculated by the G*power 3 program [24].

### Measurement of specific PCs and data processing in the validation study

In the validation study, specific phosphoglycerolipids were quantified by LC/MS (Ultimate 3000/Q-exactive [Thermofisher Scientific, Waltham, MA]). Lipid extracts were separated by the LC system using an InertSustainSwift C18 column (2.1 × 150 mm, 1.9 μm; GL Sciences, Tokyo, Japan). A binary solvent system comprising two mobile phases was used; mobile phase A consisted of acetonitrile:methanol:H_2_O (9:9:2) with 10 mM ammonium formate and 0.1% formic acid, and mobile phase B consisted of isopropanol:acetonitrile (4:1) with 10 mM ammonium formate and 0.1% formic acid. The flow rate was set at 200 μL/min and the column oven was held at 55°C. Before gradient elution, the column was equilibrated with 20% mobile phase B. Samples (5 μL) were injected, and gradient elution was initiated at 20% mobile phase B, increased to 60% from 0 to 10 min, and then increased to 100% from 10 to 12 min. Gradient elution was maintained at 100% mobile phase B for 3 min. The column was equilibrated with 20% mobile phase B for 10 min before the next sample was injected. MS detection was performed with alternation among the high mass-accuracy full scan (full MS) in the positive and negative ion modes and data-dependent MS2 scan (dd-MS2) in the negative ion mode. We used the inclusion list for dd-MS2 (targeted specific PCs) and dd-MS2 was set to Top 3. The scan resolutions for full MS and dd-MS2 were 140,000 and 17,500, respectively. The spray voltage was set to 3.5 and -2.5 kV in positive and negative ion modes, respectively. The following conditions were held constant for both the positive and negative ion modes. The scan range of the MS was set to m/z 500–1000. The plasma samples of patients with SJS/TEN-associated chronic SOCs and healthy volunteers were randomized across the run. Raw LC/MS data were processed using TraceFinder (Thermofisher Scientific), which allows detection and quantification of ion peak areas at specific m/z and RT. Full MS data in the negative ion mode were used for quantification. The estimated concentrations of PCs were determined using a curation curve constructed using one PC standard (PC[12:0/12:0] in the present study), as frequently applied in the previous studies [25,26]. The slope and goodness-of-fit values for the linear regression (R^2^) of the PC standard (PC[12:0/12:0]) were 1.001 and 0.998, respectively.

## Results

### Plasma lipid profiles of patients with SJS/TEN-associated chronic SOCs, patients with atopic dermatitis, and healthy volunteers

Plasma lipid profiling of 17 patients with SJS/TEN-associated chronic SOCs, 24 patients with atopic dermatitis, and 32 healthy volunteers were determined by lipidomic analysis using a combination of two LC/MS platforms. The analysis yielded 386 lipid molecules, including 114 phosphoglycerolipids, 47 sphingolipids, 182 neutral lipids, and 43 oxylipins (Table 2). These lipid molecules include LPC, LPE, PC, ePC, PE, ePE, PI, SM, Cer, GCer, CoQ, ChE, DG, ether-type DG (eDG), TG, ether-type TG (eTG), PUFA, COX metabolites, LOX metabolites and P450 metabolites.

**Table 2:** Results of the screening study for identification of lipid classes and number of individual lipid molecules in the plasma of patients with SJS/TEN-associated chronic SOCs, patients with atopic dermatitis, and healthy volunteers.

Lipid type	Lipid classes	Number of molecules (oxidized)
Phosphoglycerolipids (PGLs)	lysophosphatidylcholine (LPC)	8
	lysophosphatidylethanolamine (LPE)	2
	phosphatidylcholine (PC)	44 (2)
	ether-type PC (ePC)	23
	phosphatidylethanolamine (PE)	10
	ether-type PE (ePE)	18
	phsophatidylinositol (PI)	9
Sphingolipids (SLs)	sphingomyelin (SM)	34 (1)
	ceramide (Cer)	9
	glycosylceramide (GCer)	4
Neutral lipids (NLs)	coenzyme Q (CoQ)	1
	cholesterolester (ChE)	18
	diacylglycerol (DG)	17
	ether-type DG (eDG)	4
	triacylglycerol (TG)	139
	ether-type TG (eTG)	3
Oxylipins (OXLs)	Polyunsaturated fatty acids (PUFA)	3
	cyclooxygenase (COX) metabolite	5
	lipoxygenase (LOX) metabolite	21
	cytochrome P450 (P450) metabolite	14
	total	386

### Comparison of plasma lipid profiles of patients with SJS/TEN-associated chronic SOCs and healthy volunteers

**Fig 1:**
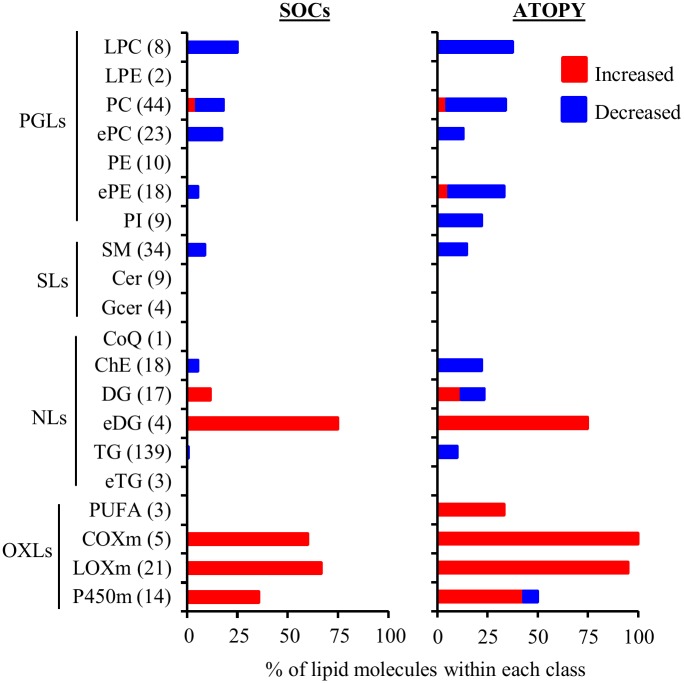
Comparison of the percentage of identified lipid molecules in patients with SJS/TEN-associated chronic SOCs and atopic dermatitis with those in healthy volunteers. The number following the lipid class indicates total number of detected lipid molecules in each lipid class. SOCs, patients with SJS/TEN-associated chronic SOCs; ATOPY, patients with atopic dermatitis. Other abbreviations are described in Table 2.

For screening of specific alterations in lipid molecules in SJS/TEN-associated chronic SOCs, we set FDR < 0.15 as the primary threshold. Overall, the levels of 47 lipid molecules (12.2% of the total number of identified lipid molecules) exceeded the primary threshold (S3 Table). The levels of 29 lipid molecules (7.5% of the total) were higher, whereas the levels of 18 lipid molecules (4.7% of the total) were lower than the threshold, in patients with chronic SOCs. The differences were inclined to specific types of lipid classes. The lipid molecules detected in higher levels in patients with chronic SOCs were dominant in DG, eDG, and oxylipins, such as PGE_2_ and 12-hydroxyeicosatetraenoic acid (12-HETE) (Fig 1). On the other hand, the lipid molecules detected in lower levels in patients with chronic SOCs were dominant in phosphoglycerolipids, such as LPC, PC, ePC, and ePE. Only oxidized PC levels, such as PC(34:2)+O and PC(36:2)+O levels, were higher in patients with chronic SOCs within the phosphoglycerolipids.

### Comparison of plasma lipid profiling between atopic patients and healthy volunteers

Once we delineated the differences in the plasma lipid profiles between patients with SJS/TEN-associated chronic SOCs and healthy volunteers, we next compared plasma lipid profiles between healthy volunteers and patients with another inflammatory disease (atopic dermatitis) to delineate specific alterations by SJS/TEN-associated chronic SOCs. Overall, the levels of 92 lipid molecules (23.8% of the total number of identified lipid molecules) exceeded the primary threshold (S4 Table). The levels of 40 lipid molecules were higher in patients with atopic dermatitis, and levels of 52 lipid molecules were lower than the threshold. The lipid molecules detected in higher levels in patients with atopic dermatitis were dominant in DG, eDG, and oxylipins (Fig 1). In addition, the lipid molecules that were decreased in patients with atopic dermatitis were dominant in phosphoglycerolipids. The overall trend of lipid alteration against that in healthy volunteers was similar between patients with SJS/TEN-associated chronic SOCs and atopic dermatitis.

### Specific lipid alterations in patients with SJS/TEN-associated chronic SOCs

**Fig 2:**
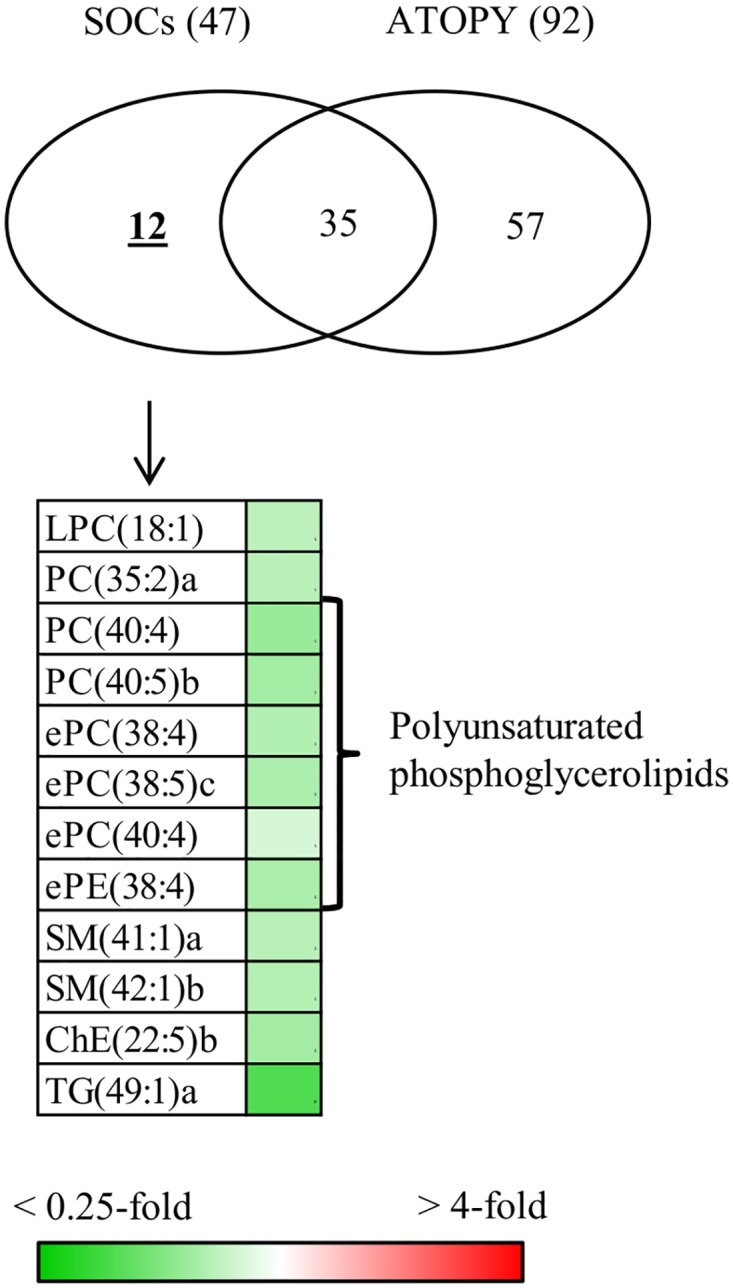
Venn diagrams of the number of identified lipid molecules that were abundant between healthy volunteers and patients with SJS/TEN-associated chronic SOCs and between healthy volunteers and patients with atopic dermatitis. The number following each subject/patient group indicates the total number of qualified lipid molecules in patients with SJS/TEN-associated chronic SOCs (SOCs) and patients with atopic dermatitis (ATOPY). Only the qualified lipid molecules between healthy volunteers and patients with SJS/TEN-associated chronic SOCs are shown in the heat map. The heat map was generated using mean fold changes in the levels of molecules, calculated as ratios of values obtained for patients with SJS/TEN-associated chronic SOCs to those obtained for healthy volunteers. Other abbreviations are described in Table 2.

To further address the specific alterations by SJS/TEN-associated chronic SOCs, we first screened for the lipids altered only in SJS/TEN-associated chronic SOCs (Fig 2). Of the 47 lipid molecules putatively different between patients with SJS/TEN-associated chronic SOCs and healthy volunteers, 12 were specific for chronic SOCs, and the levels of all 12 were decreased. The specific lipid molecules were LPC(18:1), PC(35:2)a, PC(40:4), PC(40:5)b, ePC(38:4), ePC(38:5)c, ePC(40:4), ePE(38:4), SM(41:1)a, SM(42:1)b, ChE(22:5)b, and TG(49:1)a. Because half of these were polyunsaturated phosphoglycerolipids, we also characterized their fatty acid compositions. As shown in Table 3, 5 of the 6 polyunsaturated phosphoglycerolipids contained AA (20:4), and 4 of these were PC, suggesting that AA-containing PC is specifically altered in chronic SOCs.

**Table 3:** Fatty acid side chains, calculated effect size, and statistically required sample size of specific polyunsaturated phosphoglycerolipids altered in SJS/TEN-associated chronic SOCs.

Lipids	Fatty acid side chains	Effect size (d) in screening study (Healthy vs SOCs)	Required total sample size (for α = 0.05 and β = 0.1)
PC(40:4)	20:0/20:4	0.86	48
PC(40:5)b	18:0/22:5	0.82	54
ePC(38:4)	18:0e/20:4	0.83	52
ePC(38:5)c	18:1e/20:4	0.88	46
ePC(40:4)	20:0e/20:4	0.72	68
ePE(38:4)	18:0e/20:4	0.72	68

**Fig 3:**
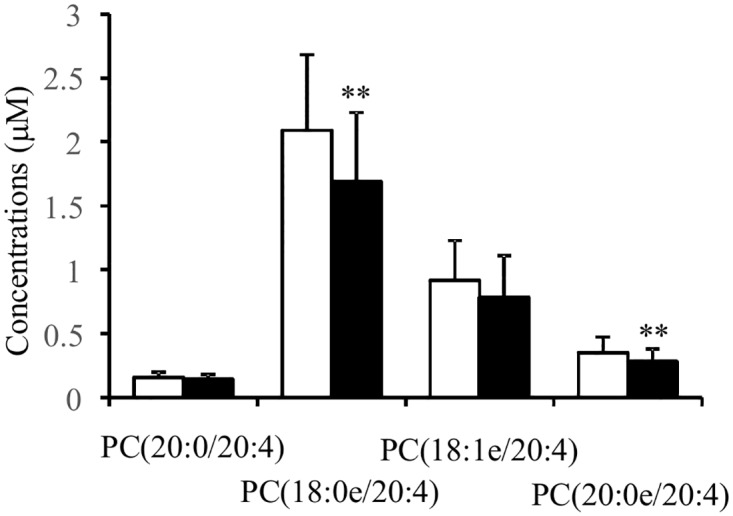
Specific changes in AA-containing PC and ePC molecules in patients with SJS/TEN-associated chronic SOCs in the validation study. Plasma concentrations of AA-containing PC and ePC molecules were determined and are reported as the mean ± SD. Statistical significance of the differences between measurements in the healthy volunteers and patients with SJS/TEN-associated chronic SOCs was assessed by Student’s *t*-test followed by Bonferroni multiple comparison collection and is reported as ** p < 0.01. Abbreviations are described in Table 2.

To further validate the specific lipid alterations in patients with SJS/TEN-associated chronic SOCs, we calculated required sample size for reliable statistical power (α = 0.05 and β = 0.1) based on effect size in the screening study. As shown in Table 3, the required total sample sizes ranged from 46 to 68, and we recruited up to 106 additional samples (55 healthy volunteers and 51 patients with SJS/TEN-associated chronic SOCs). We quantified the plasma levels of the specific lipid molecules, PC(20:0/20:4), PC(18:0e/20:4), PC(18:1e/20:4), and PC(20:0e/20:4), and compared their levels between patients with SJS/TEN-associated chronic SOCs and healthy volunteers (S5 Table). The determined plasma concentration range of PC(18:0e/20:4) was 0.62 to 3.65 μM, which was compatible with previously determined levels of serum PC(20:0/20:4) [27]. As shown in Fig 3, PC(18:0e/20:4) and PC(20:0e/20:4) were significantly decreased in patients with SJS/TEN-associated chronic SOCs. It has been well documented that plasma lipid levels differ according to gender- and/or age-associated dimorphisms [23,27,28]. Because women were dominant in subject group, we extracted their data separately and compared the plasma levels of PC(18:0e/20:4) and PC(20:0e/20:4) between healthy volunteers and patients with SJS/TEN-associated chronic SOCs. In addition, we also selected an age range (20–55 years), because lipid alteration in post-menopausal state has been reported previously [23,27]. The sample size of the female-extracted or age-selected analysis was 68 or 76, respectively, and fulfilled the required sample size determined by the screening study. As shown in Table 4, both PC(18:0e/20:4) and PC(20:0e/20:4) were significantly decreased in patients with SJS/TEN-associated chronic SOCs, even in female-extracted or age-selected subjects. These results suggest that the decreased levels of PC(18:0e/20:4) and PC(20:0e/20:4) in patients with SJS/TEN-associated chronic SOCs are not affected by gender- or age-associated dimorphisms.

**Table 4:** Plasma concentration of PC(18:0e/20:4) and PC(20:0e/20:4) in stratified subject groups of patients with SJS/TEN-associated chronic SOCs and healthy volunteers.

Lipids	Category	sample size		average		SD		Statistical value	
		Healthy	SOCs	Healthy	SOCs	Healthy	SOCs	p-value	p-value (corrected)
PC(18:0e/20:4)	all	55	51	2.09	1.69	0.59	0.54	0.000	0.002
	female	35	33	2.15	1.76	0.57	0.50	0.003	0.012
	age range 20 to 55	47	29	2.17	1.75	0.59	0.55	0.003	0.010
PC(20:0e/20:4)	all	55	51	0.35	0.28	0.12	0.10	0.001	0.006
	female	35	33	0.37	0.28	0.11	0.09	0.000	0.002
	age range 20 to 55	47	29	0.36	0.30	0.12	0.10	0.009	0.038

## Discussion

In the present study, we first demonstrated that plasma lipids are altered in patients with SJS/TEN-associated chronic SOCs. In the screening phase, our data showed that plasma levels of inflammatory oxylipins, such as PGE_2_ and 12-HETE, oxidized PCs, and eDGs are increased in patients with SJS/TEN-associated chronic SOCs, whereas phosphoglycerolipids, such as LPCs, PCs, ePCs, and ePEs, are reduced. Comparison of the results with those for patients with atopic dermatitis and determination of fatty acid side chains in phosphoglycerolipids demonstrated that AA-containing PCs and ePCs, as putative specific lipids, decreased in chronic SOCs. Finally, the decreased levels of AA-containing ePCs, PC(18:0e/20:4), and PC(20:0e/20:4) were validated using a reliable sample size and gender- or age-extracted comparison.

AA is the major polyunsaturated fatty acid, from which oxylipins, such as PGE_2_ and 12-HETE, are produced. A class of phospholipase A_2_ (PLA_2_) is responsible for the primary step to oxylipin synthesis through releasing AA from phosphoglycerolipids [29]. So far, more than 30 isoforms and related enzymes of PLA_2_ have been identified, and their substrate selectivity depends on the isoform [30]. In addition, it has been reported that several isoforms of PLA_2_, secretory group IIa, V and X PLA_2_, are associated with the ocular inflammatory response [31]. Thus, these specific isoforms of PLA_2_ might be responsible for the specific decrease of AA-containing ePCs in chronic SOCs, and identification of the specific PLA_2_ will lead to reveal the biological role of the observed changes in AA-containing phospholipids in chronic SOCs.

Recently, we demonstrated that polymorphisms in Toll-like receptor 3 (TLR3) with HLA-A*02:*06* exerted synergistic effects on SJS/TEN-associated chronic SOCs [32]. It has been reported that TLR3 activates both cytosolic and secreted PLA_2_ to increase the release of AA from AA-containing phosphoglycerolipids [33]. Thus, TLR3 polymorphisms would be associated with decreased AA-containing ePCs in chronic SOCs and this might be a key factor in the development of chronic SOCs.

Alterations in inflammatory oxylipins, such as PGE_2_ and 12-HETE, is another possible lipid alteration in SJS/TEN-associated chronic SOCs, although this observation needs to be validated by further study. PGE_2_ is primarily characterized as a suppressant of T helper type-1 (Th1) cells because of its suppressive effect on cell proliferation, differentiation, and cytokine production from Th1 cells [34,35]. However, recent reports indicate that PGE_2_ regulates Th differentiation in different directions depending on PGE_2_ receptors and their host cell types. For example, PGE_2_-PGE_2_ receptor 1 facilitates Th1 differentiation in draining lymph nodes [36], whereas PGE_2_-PGE_2_ receptor 2/4 facilitates Th1 differentiation in T cells and dendritic cells [37]. It has been reported that SJS/TEN lesions display a mixed Th1/ T helper type-2 (Th2) pattern [38]. Thus, PGE_2_ might play a double-edged role in Th1/Th2 differentiation with different types of receptors and/or effector cells in lesion of SJS/TEN associated chronic SOCs. Additional data supports an alternative role for PGE_2_. We previously reported a down-regulation of a PGE_2_ receptor 3 (EP3) in the conjunctival epithelium of SJS/TEN patients, suggesting that EP3 might help prevent ocular surface inflammation [7]. Increased production of PGE_2_ might compensate for reduced expression levels of EP3.

Oxidized PCs and eDGs were also putative lipid alterations and elevated in patients with SJS/TEN-associated chronic SOCs in our screening study. Oxidized PCs have been characterized as the major component of minimally oxidized low density lipoprotein and have been demonstrated to accumulate at sites of inflammation such as arterial lesions [39,40]. On the other hand, it has been reported that an inflammatory cytokine, interleukin-1α, selectively generates eDGs rather than DGs from phosphoglycerolipids [41]. Thus, potentially elevated plasma oxidized PCs and eDGs would also implicate the inflammatory state of chronic SOCs.

Although our results demonstrated specific lipid alterations associated with chronic SOCs in plasma, there are several limitations. First, this study applied a cross sectional design, which does not allow for the differentiation between causes and effects. Second, subjects recovered from SJS/TEN but without chronic SOCs, rather than healthy volunteers, would be more suitable as controls for chronic SOCs associated with SJS/TEN. However, patients recovered from SJS/TEN without any chronic symptoms are considered healthy. Thus, we employed healthy volunteers as the control subjects in the present study. Third, we used plasma samples from patients suffering from SJS/TEN-associated chronic SOCs to delineate the lipid profile changes. Although plasma lipid profiling has emerged as a useful tool to understand the pathophysiological alterations in specific organs, it remains unclear whether plasma lipid profiles fully reflect those in ocular tissues. However, so far, lipid profiling of ocular tissues is difficult to perform in human studies due to the invasiveness of sampling and that of lacrimal fluid is not commonly collected because of its low sample volumes. Future studies with ocular tissues and/or lacrimal fluid using very sensitive lipidomic systems would provide more insight into the pathophysiology of SJS/TEN-associated chronic SOCs. Fourth, there might be several confounding factors in plasma lipid profiling, although our results demonstrated that the decreased levels of AA-containing ePCs in chronic SOCs are unrelated with two of the major confounders, sex and age. Metabolic disorder, body mass index, and intake of oral contraceptives have been reported to affect plasma lipid levels [42–44]. In the present study, we did not collect subject information other than sex and age. However, in the previous reports, no alterations were reported for the specific lipids, PC(18:1e/20:4) and PC(20:0e/20:4), except in the presence of hypertension. In addition, other ePCs, such as ePC(32:0) and ePC(32:1), were also decreased in patients with hypertension, while they were not altered in SJS/TEN-associated chronic SOCs. Thus, the specific decrease of AA-containing ePCs in the plasma is probably a specific to patients with the chronic SOCs.

In conclusion, we characterized the plasma lipid profiles of patients with SJS/TEN-associated chronic SOCs using a lipidomics approach. By comparing lipid profiles of patients with the chronic SOCs to those of healthy volunteers, we revealed that specific alterations of the plasma lipid profiles are associated with the chronic SOCs. Moreover, we validated the decreased levels of AA-containing ePCs, PC(18:0e/20:4), and PC(20:0e/20:4) in chronic SOCs using a reliable sample size. Our study provides insight into alterations of plasma lipidomic profiles that are associated with SJS/TEN-associated chronic SOCs patients. Because our present study has demonstrated decreased ePCs as the most specific plamsa lipid alterations in the chronic SOCs, further studies addressing ePC levels as well as outcomes of restoring ePCs in eye tissues of patients with the chronic SOCs would advance to understand the ePC-related pathophysiological mechanisms that are potential therapy targets for SJS/TEN-associated chronic SOCs.

## Supporting Information

S1 Table Lipid molecules in the plasma obtained from patients with SJS/TEN-associated chronic SOCs, patients with atopic dermatitis, and healthy volunteers in the screening study. (XLSX)

S2 Table Processed data on the levels of lipid molecules in the screening study. (XLSX)

S3 Table Average value and standard deviations of the plasma levels of lipid molecules that were significantly different between patients with SJS/TEN-associated chronic SOCs and healthy volunteers (Healthy) in the screening study. (XLSX)

S4 Table Average value and standard deviations of the plasma levels of lipid molecules that were significantly different between patients with atopic dermatitis (ATOPY) and healthy volunteers (Healthy) in the screening study. (XLSX)

S5 Table Levels of arachidonic acid-containing phosphatidylcholines in the validation study. (XLSX)

## Abbreviations

12-HETE: 12-hydroxyeicosatetraenoic acid
AA: arachidonic acid
Cer: ceramide
Ch/ChE: cholesterol/cholesterol ester
CM: cold medicine
CoQ: coenzyme Q
COX: cyclooxygenase
DG: diacylglycerol
DHA: docosahexaenoic acid
eDG: ether-type DG
eLPE: ether-type lysophosphatidylethanolamine
EPA: eicosapentaenoic acid
ePC: ether-type PC
ePE: ether-type PE
EP3: PGE_2_ receptor 3
eTG: ether-type TG
GCer: glycosylceramide
iNKT: invariant natural killer T
KPUM: Kyoto Prefectural University of Medicine
LC/TOFMS: liquid chromatography-time-of-flight/mass spectrometry
LOX: lipoxygenase
LPC: lysophosphatidylcholine
LPE: lysophosphatidylethanolamine
LTB_4_-d4: tetradeuterated leukotriene B_4_
P450: cytochrome P450
PAF: platelet-activating factor
PC: phosphatidylcholine
PE: phosphatidylethanolamine
PGE_2_: prostaglandin E_2_
PI: phosphatidylinositol
PLA_2_: phospholipase A_2_
PUFA: polyunsaturated fatty acid
RT: retention time
SJS: Stevens-Johnson syndrome
SM: sphingomyelin
SOCs: severe ocular complications
TEN: toxic epidermal necrolysis
TG: triacylglycerol
Th1: T helper type-1
Th2: T helper type-2
